# Synaptic loss and amyloid beta alterations in the rodent hippocampus induced by streptozotocin injection into the cisterna magna

**DOI:** 10.1186/s42826-020-00049-x

**Published:** 2020-06-10

**Authors:** Yujin Ahn, Jincheol Seo, Junghyung Park, Jinyoung Won, Hyeon-Gu Yeo, Keonwoo Kim, Chang-Yeop Jeon, Jae-Won Huh, Sang-Rae Lee, Dong-Seok Lee, Youngjeon Lee

**Affiliations:** 1 National Primate Research Center, Korea Research Institute of Bioscience and Biotechnology (KRIBB), Cheongju, 28116 Republic of Korea; 2 Department of Functional Genomics, KRIBB School of Bioscience, University of Science and Technology (UST), Daejeon, 34113 Republic of Korea; 3 School of Life Sciences, BK21 Plus KNU Creative BioResearch Group, Kyungpook National University, Daegu, 41566 Republic of Korea

**Keywords:** Alzheimer’s disease, Streptozotocin, Cisterna magna

## Abstract

To date, researchers have developed various animal models of Alzheimer’s disease (AD) to investigate its mechanisms and to identify potential therapeutic treatments. A widely recognized model that mimics the pathology of human sporadic AD involves intracerebroventricular (ICV) injection with streptozotocin (STZ). However, ICV injections are an invasive approach, which creates limitations in generalizing the results. In this study, we produced a rodent model of AD using STZ (3 mg/kg) injection via the cisterna magna (CM) once every week for 4 weeks, and analyzed at 4 weeks and 16 weeks after final injection. In the CM-STZ rodent model of AD, we observed increase in extracellular amyloid-beta (Aβ) deposition and decrease and abnormal morphology of post-synaptic protein, PSD95 in 16 weeks STZ-injected group. The model developed using our less-invasive method induced features of AD-like pathology, including significantly increased extracellular amyloid-beta deposition, and decreased synaptic protein in the hippocampus. These findings supporting the success of this alternative approach, and thus, we suggest this is a promising, less invasive model for use in future AD research.

## Introduction

Alzheimer’s disease (AD) is the most common neurodegenerative disorder causing dementia [1]. AD is pathologically characterized by amyloid-beta (Aβ) plaques, neuronal loss, and cognitive impairment [2]. Considerable research has been performed to develop AD models and conduct preclinical studies to investigate the mechanisms underlying AD and potential treatment options [3].

Streptozotocin (STZ) is a diabetogenic compound able to induce insulin-resistant cells similar to sporadic AD neural cells [4]. Therefore, intracerebroventricular (ICV) injection with STZ is used to mimic the pathology of human sporadic AD [5]. However, a major disadvantage of this method is the invasiveness of the ICV injection, which involves craniotomy and directly damages the brain tissue. A previous paper confirmed accurate and reproducible access to the artificial cerebrospinal fluid (aCSF) of rodents using a cisterna magna (CM) injection method [6]. Furthermore, the injected molecules diffused into the parenchyma, similar to diffusion following ICV injection [7].

In the present study, we produced a rodent model of AD, using STZ injection via the CM and determined the development of AD-like pathologies. It was found that this model successfully induced AD-like pathological features, such as extracellular Aβ accumulation and synaptic loss.

## Materials and methods

### Experimental animals

Male Sprague Dawley rats (460 ± 20 g, 14 weeks old) were housed in a temperature-controlled room (20–23 °C) with 30–60% humidity in a 12-h light-dark cycle with ad libitum access to standard food pellets and water. All procedures were approved by the Korea Research Institute of Bioscience and Biotechnology Institutional Animal Care and Use Committee (Approval No. KRIBB-AEC-18016).

### CM injection of STZ and brain sampling

**Fig. 1:**
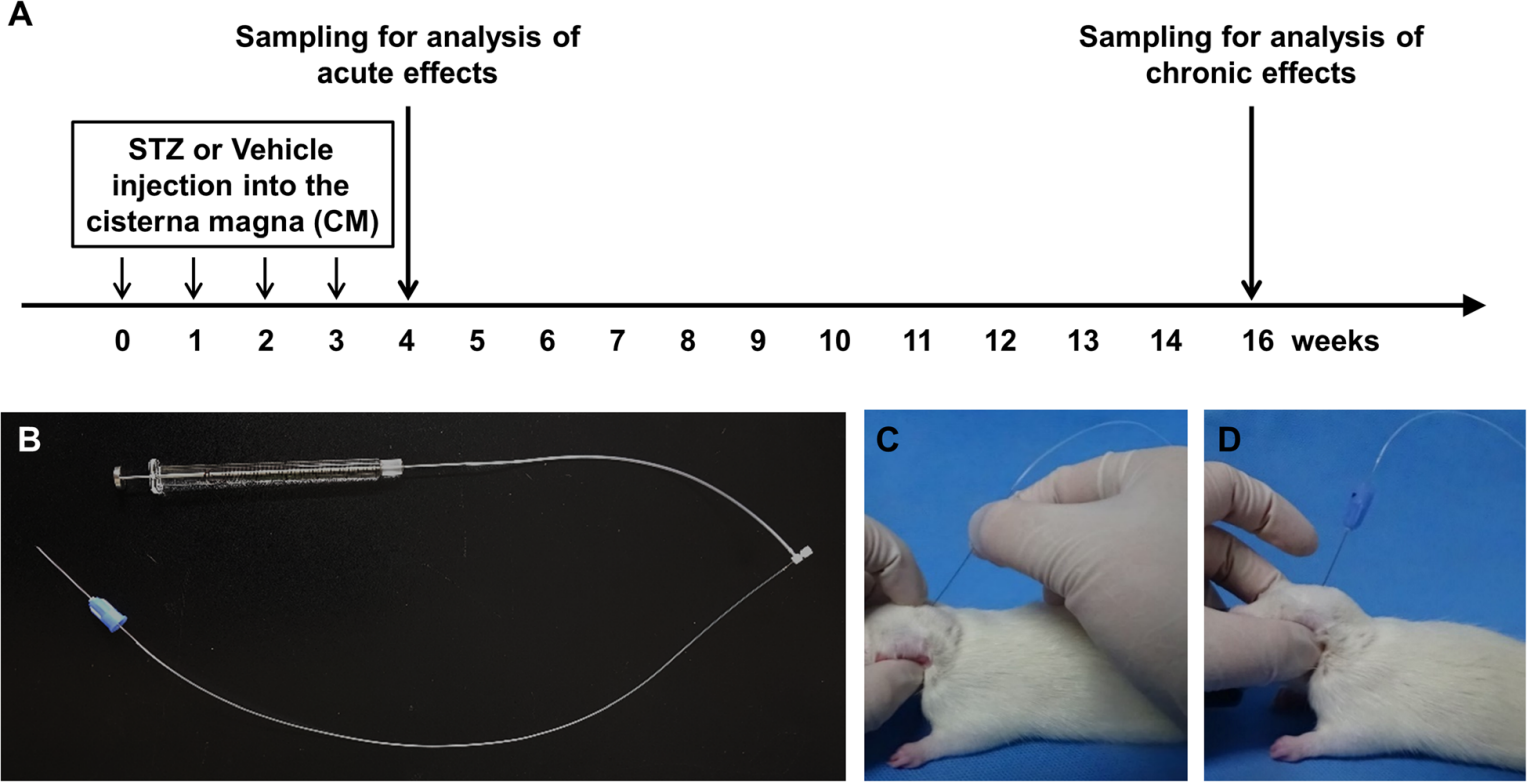
Experimental design and cisterna puncture method. **a** A schematic timeline showing CM-STZ injections and subsequent sampling points. **b** The needle tubing assembly for CM injections. A 27G dental needle was connected with polyethylene tubes and the terminal of the tube was linked to 22G Hamilton syringe. **c-d** The method used for CM injections

For CM injection of STZ, we used a needle-tubing assembly that comprised of PE10 and PE50 tube tubing, 27G dental needle, PE10/PE50 tubing connector and 22G Hamilton syringe (Fig. 1b) The rats were anesthetized with 3% isoflurane in an induction chamber and maintained under isoflurane anesthesia at 2% during injection. STZ (Sigma-Aldrich, St. Louis, MO, USA) was dissolved in aCSF and was infused into the CM at a rate of 10 μl / min (10 μl total volume, final dose 3 mg/kg). The needle was maintained in place for 1 min after injection. Rats received injections once every week for 4 weeks, before being sacrificed for analysis at acute (4 weeks) and chronic (16 weeks) time points (Fig. 1a). For tissue sampling, rats were anesthetized with 30% urethane and transcardially perfused with phosphate-buffered saline (PBS). The brain was dissected, and the hemisphere was used for immunohistochemistry and western blot analysis, respectively.

### General health monitoring

Body weight was recorded weekly prior to injections. Blood glucose levels were measured using Accu-Chek® Guide meter (Roche, Basel, Switzerland) on the 4 and 16 weeks after the first STZ injection.

### Immunohistochemistry

The hemisphere was fixed in 10% neutral buffered formalin for 2 days at 4 °C. After dehydrating with 30% sucrose, the tissues were embedded in optimal cutting temperature compound, and transverse sections (30 μm) were serially cut using a cryostat. The sections were then placed in 88–91% formic acid for antigen retrieval of Aβ. After blocking using 4% normal horse serum for 2 h, sections were incubated overnight at 4 °C in diluted primary antibody for Aβ (6E10; Novus, Plainsboro, NJ, USA) and PSD95 (Abcam, Cambridge, MA, USA). Sections were then incubated with biotinylated anti rabbit-IgG secondary antibody (Vector Laboratories, Burlingame, CA, USA). The sections were stained with DAB (3,3′-Diaminobenzidine; Vector Laboratories).

### Western blot analysis

The hippocampus was harvested from the hemisphere and homogenized in RIPA buffer (Thermo Scientific, Waltham, MA, USA). Proteins lysates were loaded onto 10–16% SDS-PAGE gels, transferred to nitrocellulose membranes and blocked with blocking buffer (BD Biosciences, Franklin Lakes, NJ, USA). Membranes were incubated at 4 °C overnight with primary antibodies against PSD95 (Abcam) and β-actin (Sigma-Aldrich). Following washing with TBST (tris-buffered saline with 0.1% tween 20), the membranes were incubated with secondary antibodies (Cell Signaling, Danvers, MA, USA) for 1 h at room temperature. Specific binding was detected using a chemiluminescence detection system (Bio-Rad, Hercules, CA, USA).

### Statistical analysis

The data represent the mean and standard deviation (SD) from three independent experiments (*n* = 3). Statistical significance was determined using two-way analysis of variance (ANOVA) conducted by GraphPad Prism 5 software (San Diego, CA, USA).

## Results

### Change in body weight and blood glucose by CM-STZ

**Fig. 2:**
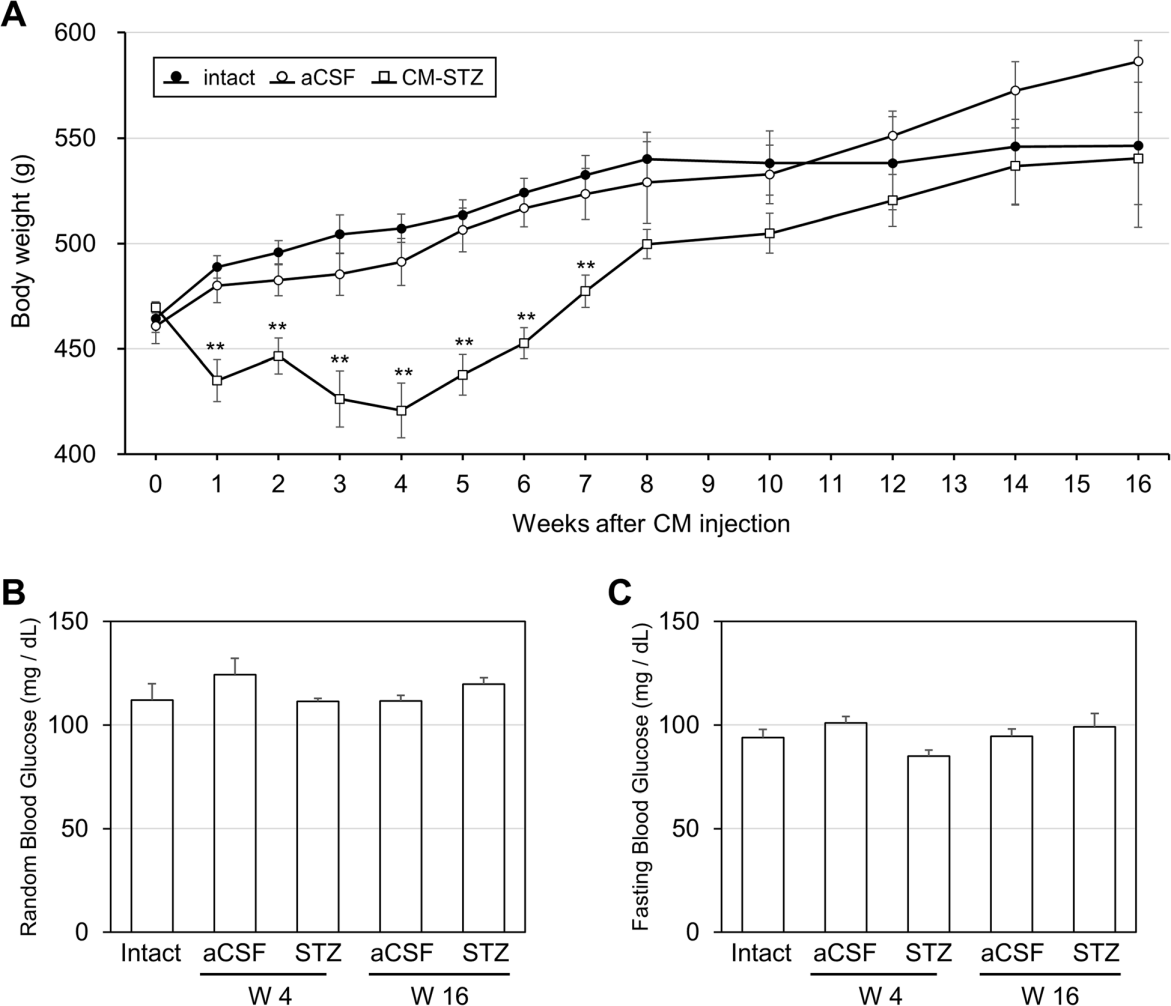
Change in body weight and blood glucose level by STZ. **a** Body weight was monitored throughout the experimental period. **b-c** The level of blood glucose was measured at random or in a fasting state at week 4 or 16. The data are presented as mean values ± SD (*n* = 3). ** denotes *p* < 0.01

Body weight was reduced in the STZ-injected group when compared to the aCSF-injected group until 4 weeks. However, after the final injection, body weight appeared to recover, with no significant difference between the STZ-injected group and control animals by 8 weeks (Fig. 2a). To analyze the effect of STZ on metabolism, we measured blood glucose level at random or in a fasting state at week 4 and 16. There was no significant alteration in the average blood glucose level between aCSF- and STZ-injected groups (Fig. 2b and c).

### CM-STZ induced extracellular Aβ accumulation and synaptic loss

**Fig. 3:**
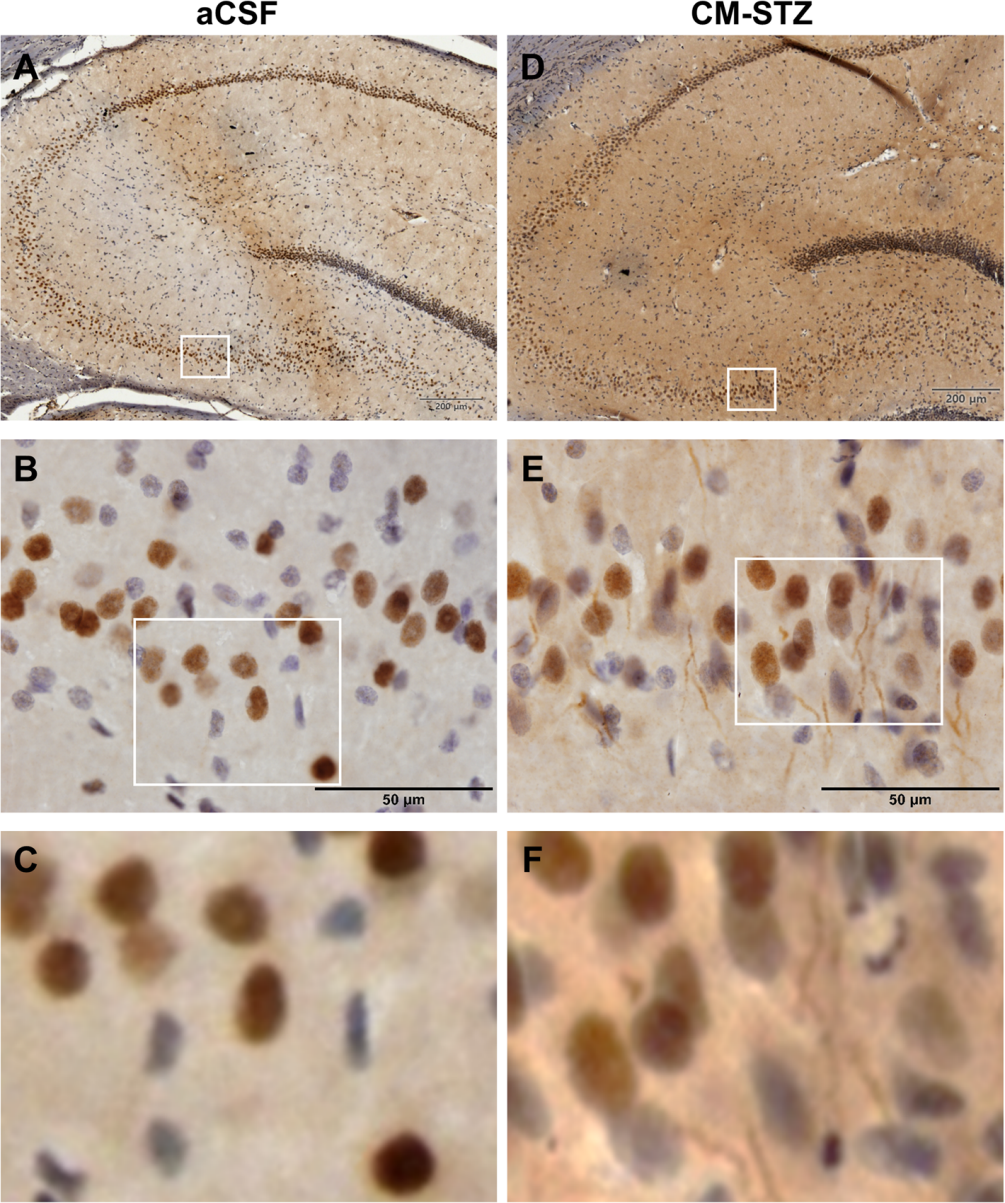
Extracellular Aβ in the hippocampal CA3. Representative sections showing Aβ (6E10) expression (brown) in area CA3 of the hippocampus from aCSF-injected (left; **a-c**) and STZ-injected (right; **d-f**) animals. Counterstaining with hematoxylin can be seen in blue. Scale bars = 50 μm. The lower panels show high-magnified images of the regions indicated by white square in the upper panels, respectively

**Fig. 4:**
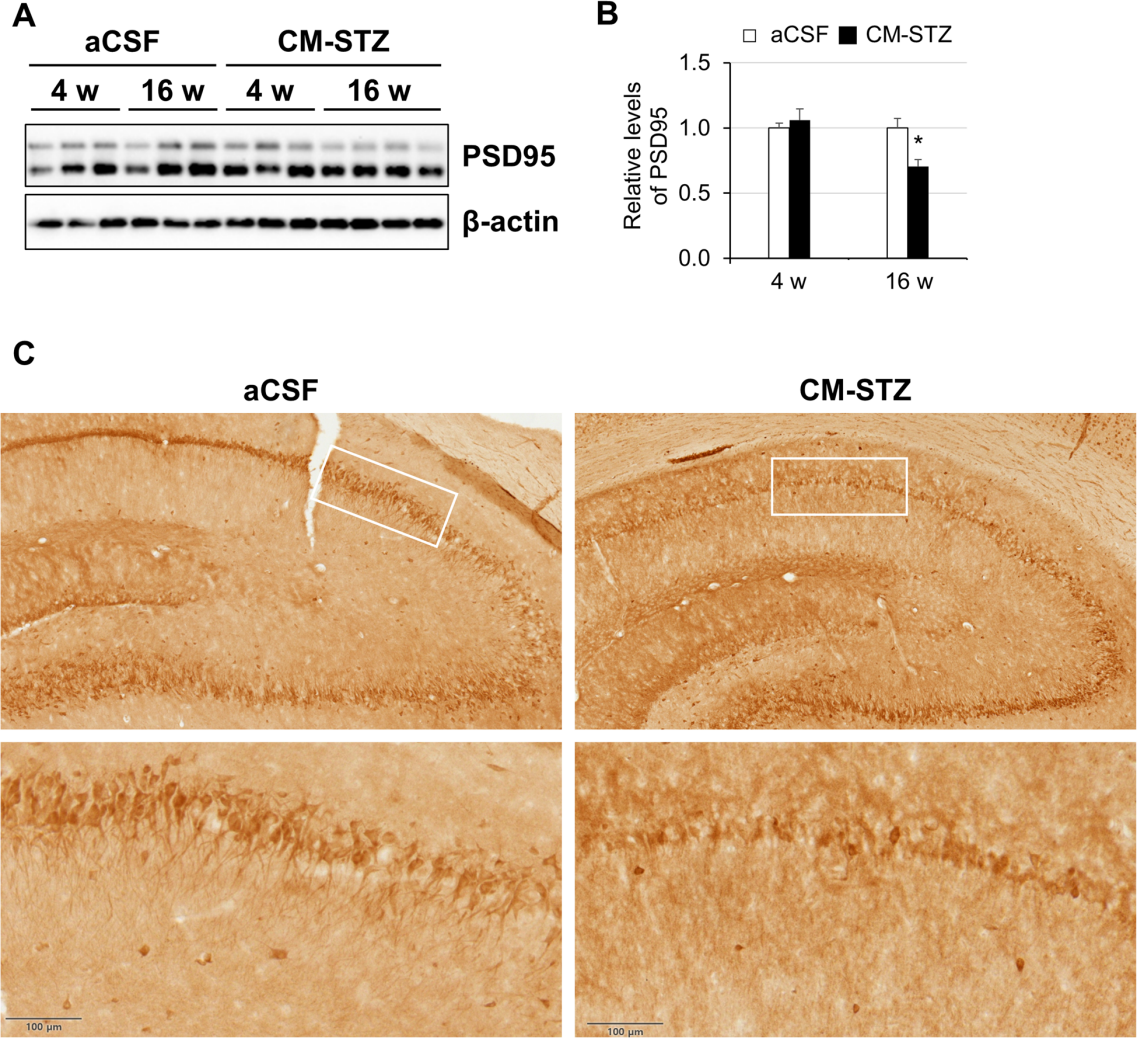
STZ-induced alteration of neuronal synapses in the hippocampal CA1. **a-b** Expression of PSD95 protein were analyzed by western blots using brain tissue from aCSF- and STZ-injected animals. **c** Representative sections showing PSD95 expression in CA1 of the hippocampus from CSF-injected (left) and STZ-injected (right) animals. The lower panels show high-magnified images of the regions indicated by white square in the upper panels. Scale bar = 100 μm. The data are presented as mean values ± SD (*n* = 3). * denotes *p* < 0.05

To confirm the presence of Aβ accumulation in CM-STZ model, we performed immunochemistry using an Aβ (6E10) antibody in CA3 of the hippocampus. Aβ accumulation was observed outside of the cells and was increased in the STZ-injected group at 16 weeks (Fig. 3). In addition, we analyzed the expression of the post-synaptic protein PSD95 by western blotting and immunohistochemistry. Although expression levels of PSD95 in the hippocampus were not altered at 4 weeks in the STZ-injected group, a significant decrease was observed at 16 weeks when compared to controls (Fig. 4a and b). Moreover, the morphology of synapse was abnormal in STZ-injected group (Fig. 4c). These results suggest that AD-like pathologies were induced by CM-STZ injection.

## Discussion

A common model of sporadic AD involves a ICV-STZ injection [8]. However, ICV injection is an invasive procedure involving skin incision and trauma to brain tissue, which can bring the reliability of results into question. Therefore, we proposed that CM injection would be equally effective at inducing AD and overcome some limitations of the ICV model. The current study provides evidence that using the CM as an alternative site of STZ injection is indeed effective at producing key pathological features of sporadic AD in a less invasive manner.

One key pathological finding was the increase in extracellular Aβ and synaptic loss in the CM-STZ model. A previous study has shown that intracellular Aβ accumulation is increased 3 months after ICV-STZ injection, and that Aβ plaques are detectable at 6 months [9]. We did not see plaques in our CM-STZ model, however our study only extended to 4 months post-STZ injection and we did observe an increase in extracellular Aβ at this time point.

Furthermore, PSD95 was reduced in the hippocampus by CM-STZ injection after 16 weeks, but not at 4 weeks. Decreases in the expression of post synaptic protein PSD95 are associated with a decline in learning and memory function [10], so this corresponds to pathological changes associated with the development of AD in our model.

Loss of body weight is a common characteristic in AD patients and ICV-STZ rodents have also been shown to lose weight without experiencing any change in blood glucose [11]. In our study, CM-STZ caused weight loss during the injection period, which recovered to control levels following the last injection, with no alteration in blood glucose.

An additional benefit of CM-STZ injection model is the ability to administer many injections over time, due to the reduced invasiveness of the procedure. In our case, we were able to repeatedly inject STZ into aCSF for 4 weeks. This approach mitigated the effects of STZ clearance during aCSF turnover.

In conclusion, we have demonstrated that our CM-STZ injection method is less-invasive and induces a similar sporadic AD-like pathology to the ICV-STZ injection model.

## Conclusions

We suggest that the method for producing a sporadic AD-like rodent model using less-invasive STZ injection via the CM.

## Data Availability

All data produced or analyzed in the present study are available upon reasonable request.

## Abbreviations

aCSF: Artificial cerebrospinal fluid
AD: Alzheimer’s disease
Aβ: Amyloid-beta
CM: Cisterna magna
ICV: Intracerebroventricular
DAB: 3,3′-Diaminobenzidine
SDS-PAGE: Sodium dodecyl sulfate–polyacrylamide gel electrophoresis
RIPA buffer: Radioimmunoprecipitation assay buffer
STZ: Streptozotocin
TBST: Tris buffered saline with 0.1% Tween 20
